# Epigenetic upregulation of *HOXC10* in non-small lung cancer cells

**DOI:** 10.18632/aging.103597

**Published:** 2020-07-19

**Authors:** Miao Li, John Simon Alsager, Zhaokai Wang, Lin Cheng, Bin Shan

**Affiliations:** 1 Department of Microbiology and Parasitology, College of Basic Medical Sciences, China Medical University, Shenyang, China; 2 Department of Biomedical Sciences, Elson S. Floyd College of Medicine, Washington State University, Spokane, WA 99202, USA; 3 The First Clinical Department of China Medical University, Shenyang, China

**Keywords:** HOXC10, cytosine methylation, non-small cell lung cancer, G-quadruplex, CpG island

## Abstract

The homeobox genes (HOX) have emerged as a new family of master regulators of development and cancer. In the current study, we examined the expression and function of *HOXC10* in human non-small cell lung cancer (NSCLC). We observed increased expression of *HOXC10* in the more aggressive human NSCLC cell line NCI-H23 over the well differentiated A549 cells. To elucidate the expression and function of *HOXC10* in NSCLC cells, we employed RT-PCR, immunoblotting, methylation-specific PCR, apoptosis assays, and xenograft model. Overexpression of *HOXC10* in A549 cells conveyed increased proliferation, reduced apoptosis, and accelerated tumor growth when transplanted into nude mice. In contrast, siRNA-mediated knockdown of *HOXC10* in NCI-H23 cells reduced proliferation and increased apoptosis. Our results further indicated that hypomethylation of the CpG island in the *HOXC10* promoter was critical to elevated expression of *HOXC10* in NSCLC cells. Lastly, we identified a G-quadruplex in the *HOXC10* promoter and its G-quadruplex formation was required for elevated expression of *HOXC10* in NSCLC cells. Moreover our results suggest that disruption of G-quadruplex formation can silence *HOXC10* expression in NSCLC cells. In summary, we report *HOXC10* as a novel tumor promoting oncogene in NSCLC cells.

## INTRODUCTION

Lung cancer has the highest incidence and mortality among all types of cancers. Non-small cell lung cancer (NSCLC) is the most common clinical subtype of lung cancer and lung adenocarcinoma is the most common histological subtype in NSCLC [1]. Despite recent advances in cancer therapeutic strategies, patients with NSCLC exhibit a poor 5-year survival rate (approximately 15%) [2], because it is usually diagnosed at an advanced stage and accompanied with aggressive local invasion, regional lymph node and distant metastasis [3]. Therefore, novel and effective therapies against lung cancer are still urgently needed.

Homeobox genes (HOX) have emerged as a novel class regulators of lung cancer. In particular, *HOXC10* has been reported to promote progression of various types of cancer, such as breast cancer [4]. Homeobox genes encode transcription factors that bind to the promoters of various target genes through their homeodomain and play critical roles in cell differentiation and embryonic development [5]. In humans, there are four HOX clusters (A-D) located on four chromosomes (7, 17, 12 and 2, respectively). *HOXC10* is a member of the *HOXC* cluster and contributes to the development of several types of cancers, including glioma, breast cancer, osteosarcoma, and thyroid cancer [6–9]. However, little is known about *HOXC10*’s expression and function in lung cancer. Methylation of cytosine in CpG dinucleotides within CpG islands located in gene promoters is a well-characterized epigenetic modification that regulates gene expression and consequently many cellular processes, including development and tumorigenesis [10]. Previous studies have shown that CpG islands are often enriched with G-quadruplex-forming sequences (G4s) [11]. G4s are stacks of G-quartets linked by loops, and the classical G4 forming sequence motifs comprise four tracts of two or more consecutive Gs separated by loop nucleotides. G4s play a crucial role in gene transcription and regulation. For example, G4 formation within the promoters of several oncogenes has been shown to suppress transcription of oncogenes such as *c-MYC*, *KRAS*, *BCL-2*, and *WNT1* genes [12–15]. However, several studies also revealed that G-quadruplex structures in promoters up-regulated gene expression, such as *RELAXIN* and *OCT4* [16, 17].

In this study, we assessed the expression of *HOXC10* in human NSCLC cell lines and analyzed the functions of *HOXC10* in tumor progression in vitro and in vivo. In addition, we focused on the molecular mechanisms that mediate upregulation of *HOXC10* expression in NSCLC cells. We examined role of cytosine methylation and the formation of G-quadruplex in the *HOXC10* promoter in upregulating *HOXC10* gene expression. Our data collectively indicate an important role for *HOXC10* in NSCLC cells and suggest *HOXC10* as a potential therapeutic target.

## RESULTS

### Elevated expression of *HOXC10* in lung adenocarcinoma cells

**Figure 1:**
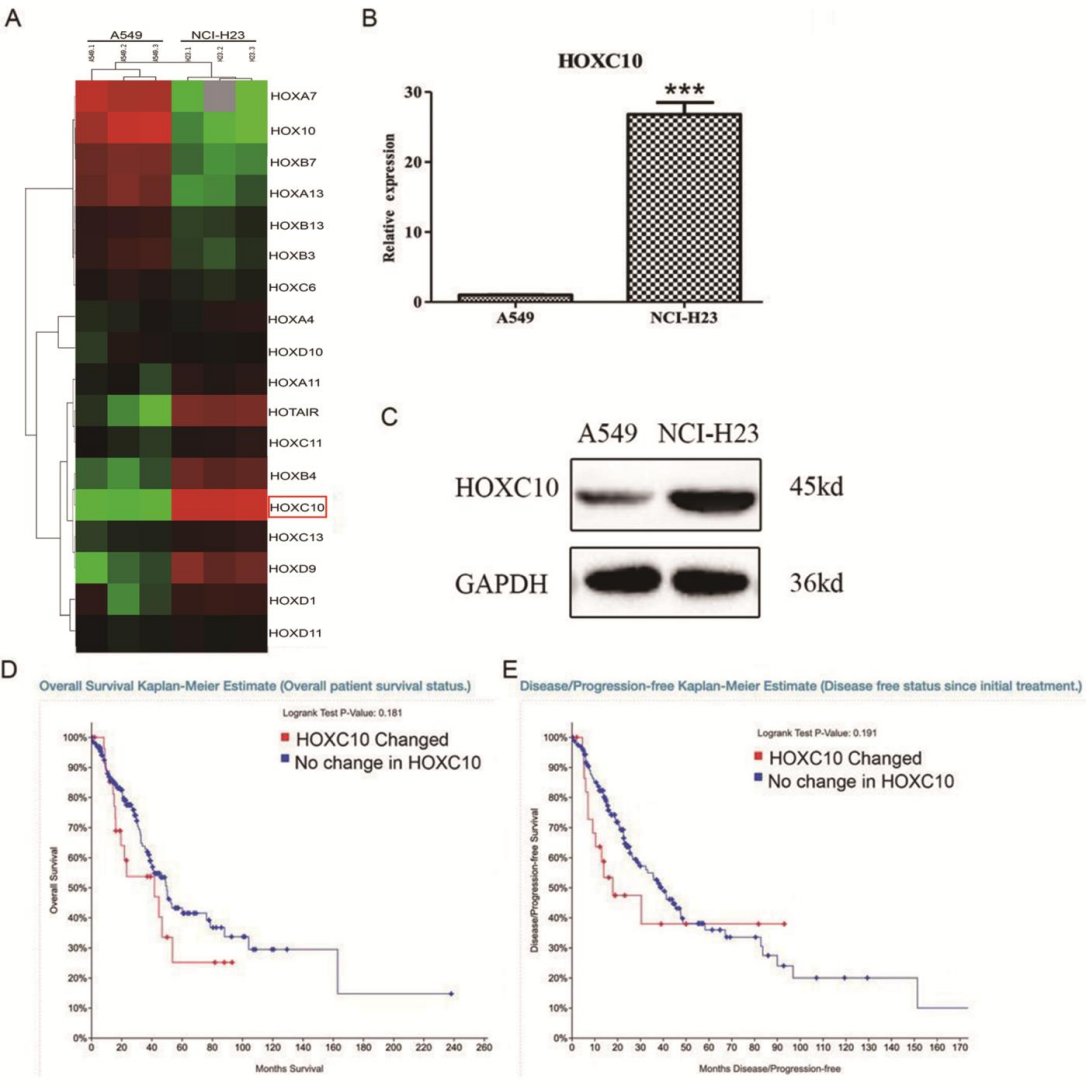
**Expression of *HOXC10* in lung adenocarcinoma cells.** (**A**) Total cell RNA was extracted from A549 and NCI-H23 cells. The transcriptomes from A549 and NCI-H23 cells were obtained and compared using RNA-SEQ. The differentially expressed HOX genes were illustrated in heatmap. *HOXC10* was marked by a red rectangle. (**B**) Similar to part A except that the RNA levels of *HOXC10* were measured and compared between A549 and NCI-H23 cells using qRT-PCR. A fold change was obtained by normalizing to the housekeeping gene RPLP0 and setting the values from the DMSO control group to one. (**C**) Total protein was extracted from A549 and NCI-H23 cells. Immunoblotting was used to assess and compare the protein levels of *HOXC10* between A549 and NCI-H23 cells. GAPDH was measured as a loading control. (**D**) Overall survival rate was compared between the patients with increase in *HOXC10* expression and the rest patients in the TCGA Lung Adenocarcinoma cohort. (**E**) Diseases free status was compared between the patients with increase in *HOXC10* expression and the rest patients in the TCGA Lung Adenocarcinoma cohort. When presented, means and standard deviations were obtained from at least 3 independent experiments. *** indicates a P value < 0.001.

We profiled transcriptomes of two human NSCLC cell lines, A549 that is well-differentiated with type II alveolar features, and NCI-H23 that is aggressive with mesenchymal features. A549 cells are abundant with epithelial markers and lack invasive ability, whereas NCI-H23 cells are low in epithelial markers and high in mesenchymal makers as reported by others and shown in our own RNA-SEQ data [18, 19]. The differentially expressed genes between two cell lines were defined as the genes with a greater than 2-fold difference and a false discovery value smaller than 0.01 as determined by EbSeq. We noticed that the differentially expressed genes included a large number of HOX genes that are critical regulators of development and cancer (Figure 1A) [20]. *HOXC10* drew our attention because its expression was higher in the aggressive NCI-H23 cells than the well-differentiated A549 cells. Moreover, a recent report suggests it regulates cell death and sensitive to therapy in breast cancer [21]. We confirmed the robust expression of *HOXC10* in NCI-H23 and modest expression of *HOXC10* in A549 cells by qRT-PCR and immunoblotting (Figure 1B, 1C). We then interrogated the expression of *HOXC10* in the RNA-SEQ data set of the TCGA Lung Adenocarcinoma cohort. we applied the default parameters set by cBIOPORTAL. “Elevated expression” and “No change” are defined based on whether *HOXC10* expression is higher than 2 x Z scores in a sample. The patients with elevated expression of *HOXC10* as defined by a Z-score greater than 2 using cBioportal analysis exhibited a trend of shorter overall survival and shorter disease free status than the patients without elevation of *HOXC10*, although the difference is slightly short of reaching statistical significance (Figure 1D, 1E).

### Promotion of cell growth by overexpression of *HOXC10* in A549 cells

**Figure 2:**
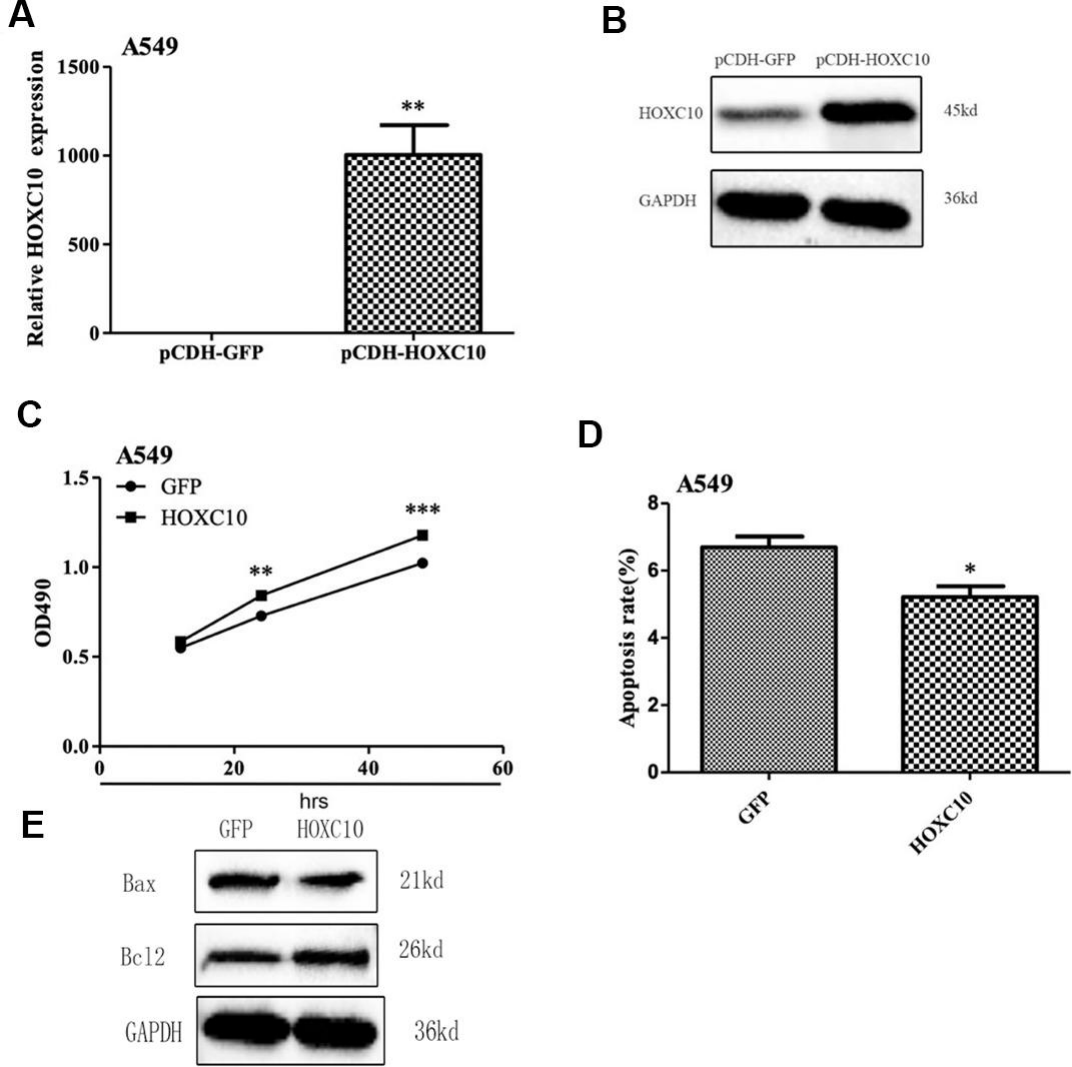
**Promotion of cell growth by overexpression of *HOXC10* in A549 cells.** (**A**) A549 cells that stabily overexpress *HOXC10* (pCDH_HOXC10) or control (pCDH_GFP) were generated as described in Methods. Total RNA was extracted and RNA levels of *HOXC10* were assessed using qRT-PCR. A fold change was obtained by normalizing to the house keeping gene *GAPDH* and setting the values from the negative control pCDH_GFP to one. (**B**) Similar to part A except that the protein levels of *HOXC10* were assessed using immnoblotting. (**C**) Similar to part A except that cell viability was measured using MTT assays. A fold change of the MTT values was obtained by setting the values from the negative control pCDH_GFP to one. (**D**) Similar to part A except that apoptosis was measured using Annexin V-FITC. Percentage of apoptotic cells were compared among the groups. (**E**) The expression of apoptosis related proteins in A549 cells with pCDH_HOXC10 or control transfected was measured by western blot analysis. When presented, means and standard deviations were obtained from at least 3 independent experiments. *, **, and *** indicate a P value < 0.05, 0.01, and 0.001, respectively.

To determine whether *HOXC10* plays a critical role in cell viability, we overexpressed *HOXC10* in the *HOXC10* low A549 cells by inserting the human *HOXC10* gene coding region into the pCDH backbone vector and transducing A549 cells with either the *HOXC10* overexpressing lentiviral vector or the backbone control. We confirmed successful overexpression of *HOXC10* by qRT-PCR and immunoblotting (Figure 2A, 2B). We then compared cell viability between control and *HOXC10* overexpression A549 cells using MTT assays. As expected, the *HOXC10* overexpressing A549 cells exhibited an 15% increase in cell viability over the control A549 cells as indicated by MTT assays (Figure 2C). We then questioned whether increased cell viability shown in MTT assays was associated with a decrease in apoptosis. We measured apoptosis using Annexin V-FITC Apoptosis Detection Kit. Indeed, overexpression of *HOXC10* caused a 22% decrease in apoptosis (Figure 2D). Next, we examined the levels of several apoptosis associated proteins by western blot. As shown in Figure 2E, the protein level of Bax was decreased in A549 cells upon *HOXC10* overexpression when compared with vector control A549 cells, whereas the protein level of Bcl-2 increased. These results suggest that overexpression of *HOXC10* is sufficient to convey increased cell viability in the *HOXC10* low A549 cells.

### Inhibition of cell growth by knockdown of *HOXC10* in NCI-H23 cells

**Figure 3:**
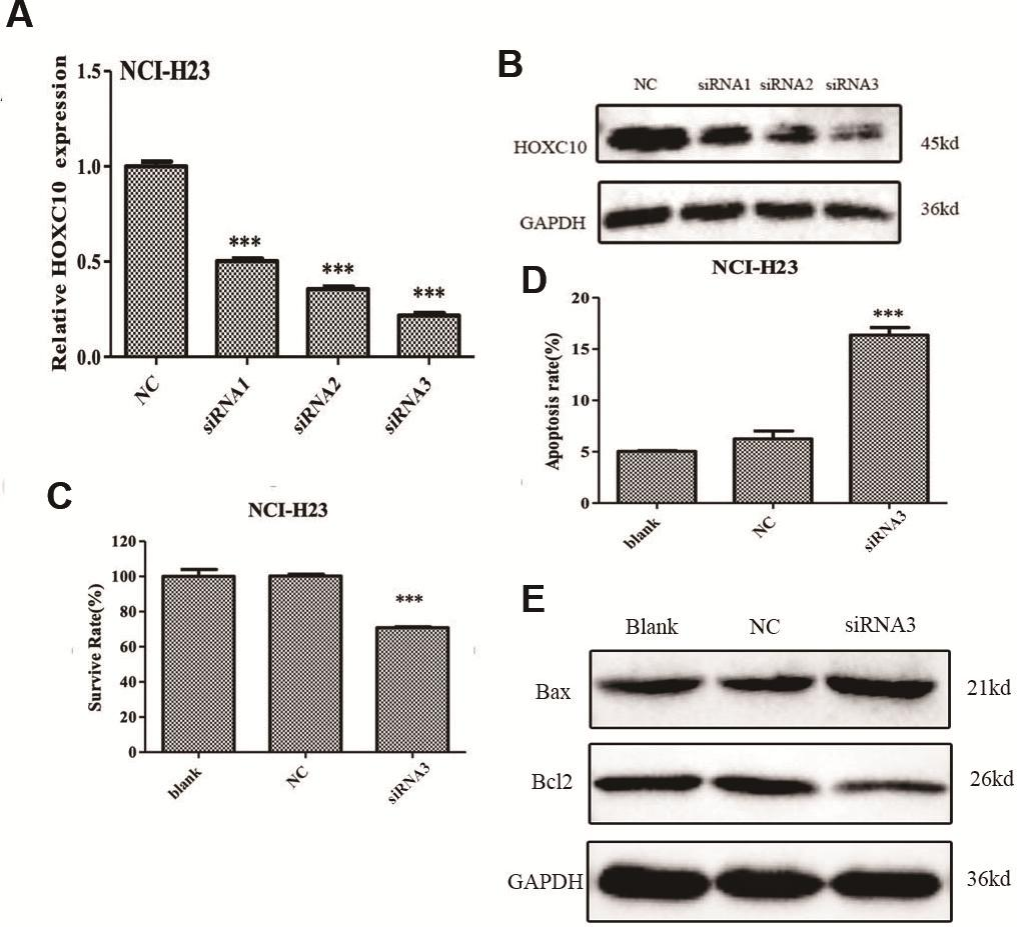
**Inhibition of cell growth by knockdown of *HOXC10* in NCI-H23 cells.** (**A**) The HOXC10-specific siRNAs (siRNA 1-3) or negative control siRNA (NC) were transfected into NCI-H23 cells. Total RNA was extracted and RNA levels of *HOXC10* were assessed using qRT-PCR. A fold change was obtained by normalizing to the house keeping gene *GAPDH* and setting the values from the negative control siRNA (NC) transfected group to one. (**B**) Similar to part A except that the protein levels of *HOXC10* were assessed using immnoblotting. (**C**) Similar to part A except that cell viability was measured using MTT assays. A fold change of the MTT values was obtained by setting the values from the negative control siRNA (NC) transfected group one. (**D**) Similar to part A except that apoptosis was measured using Annexin V-FITC. Percentage of apoptotic cells were compared among the groups. (**E**) The expression of apoptosis related proteins in NCI-H23 cells with HOXC10-specific siRNA3 or negative control siRNA transfected was measured by western blot analysis. When presented, means and standard deviations were obtained from at least 3 independent experiments. *** indicates a P value < 0.001.

We also transfected the siRNA targeting *HOXC10* mRNA into *HOXC10* high NCI-H23 cells. As illustrated in Figure 3A, 3B, the mRNA and protein levels of *HOXC10* were remarkably decreased in the siRNA3 transfected groups as compared to the control groups. Furthermore, MTT assay showed that knockdown of *HOXC10* significantly decreased cell viability by 30% in NCI-H23 cells compared with the control groups (Figure 3C). We also performed Annexin V-FITC assay to evaluate the function of *HOXC10* on apoptosis in NCI-H23 cells. As shown in Figure 3D, flow cytometry analysis revealed that knockdown of *HOXC10* in NCI-H23 cell induced a 61.8% increase in apoptosis when compared with the control groups (Figure 3D). Meanwhile, Knockdown of *HOXC10* led to decreased protein level of Bcl-2 and increased protein level of Bax in NCI-H23 cells as compared with control cells (Figure 3E).

### Promotion of tumor growth in mice by overexpression of *HOXC10* in A549 cells

**Figure 4:**
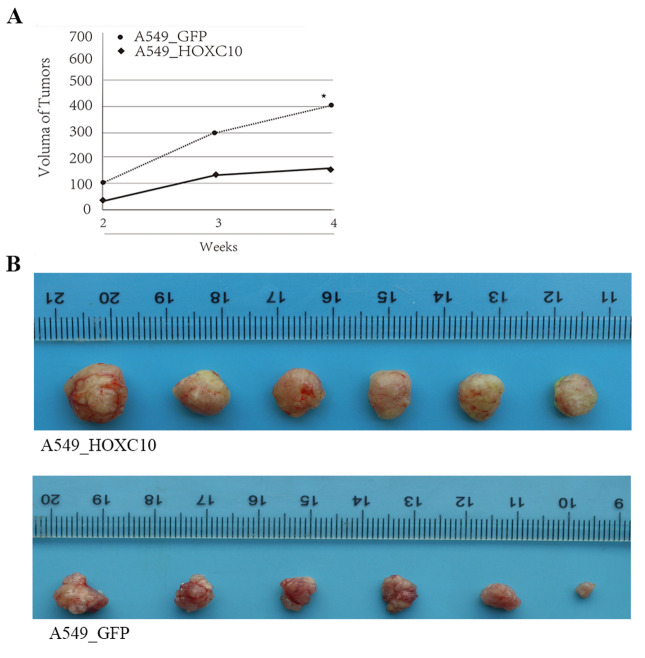
**Promotion of tumor growth in mice by overexpression of *HOXC10* in A549 cells.** (**A**) A549 cells that stably overexpress *HOXC10* (A549_HOXC10) or control (A549_GFP) were grafted into the flank of nude mice as described in the section of Materials and Methods. Volumes of the tumors formed by A549_HOXC10 and A549_GFP monitored from week 2 to week 4. Growth curves were illustrated. (**B**) The tumors were dissected and recorded on week 4 post inoculation. * indicates a P value < 0.05.

We then determined whether *HOXC10* regulates growth of NSCLC cells in vivo. The A549 variant with stable overexpression of *HOXC10* as demonstrated in Figure 2 and the backbone control variant were implanted into nude mice. Tumor growth were monitored weekly by measuring the volume and compared to the backbone control. Indeed, the *HOXC10* overexpressing A549 tumors exhibited accelerated growth when compared to the control group (Figure 4A). The size difference in tumors between two groups were further illustrated in the tumors dissected from the implanted mice (Figure 4B). These findings suggest that *HOXC10* promote growth of NSCLC cells in vitro and in vivo.

### Epigenetic regulation of *HOXC10* expression in NSCLC cells

**Figure 5:**
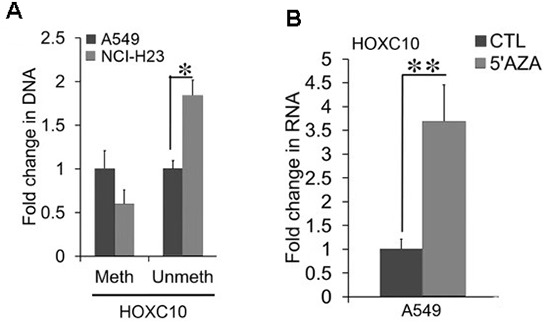
**Association between CpG methylation and the expression of *HOXC10*.** (**A**) Total cell DNA was extracted from A549 and NCI-H23 cell cultures. Cytosine methylation in the *HOXC10* CpG island was compared between two culture conditions using bisulfite treatment coupled with methylation-specific qPCR. A fold change of the methylated and unmethylated PCR products were obtained by setting the values from 2D culture to one. (**B**) Total cell RNA was extracted from A549 cells exposed to either a DNMT inhibitor, 5-Aza-CdR (1 μM) or DMSO for 72 hrs. The RNA levels of *HOXC10* were measured using qRT-PCR. A fold change was obtained by normalizing to the housekeeping gene RPLP0 and setting the values from the DMSO group to one. When presented, means and standard deviations were obtained from at least 3 independent experiments. * and ** indicate a P value < 0.05 and 0.01, respectively.

We aimed to understand how *HOXC10* is up-regulated in NSCLC cells. We noticed a CpG island that spans through the promoter, first exon, and first intron of the *HOXC10* gene. Cytosine methylation in gene promoters has been linked to transcription repression [22]. Thus, we searched for a link between *HOXC10* expression and cytosine methylation in the *HOXC10* promoter associated CpG island from the the Cancer Cell Line Encyclopedia (CCLE) data set. We interrogated Methylation SEQ data from CCLE. We compared methylation profiles in the *HOXC10* CpG island between A549 and NCI-H23 cells. Indeed, the *HOXC10* CpG island exhibited hypermethylation in A549 cells relative to that in NCI-H23 (Supplementary Figure 1). Thus, an inverse correlation between *HOXC10* expression and methylation was observed in A549 and NCI-H23 cells. To validate inverse correlation between methylation and expression of *HOXC10* in A549 and NCI-H23 cells, we employed methylation-specific PCR to assess cytosine methylation in the *HOXC10* associated CpG island [23]. The methylation-specific PCR products of the *HOXC10* CpG island identified by BS-SEQ was around 50% in NCI-H23 cells of that in A549 cells (Figure 5A). In contrast the unmethylation-specific PCR products were higher in NCI-H23 cells than in A549 cells (Figure 5A). We then questioned whether inhibition of cytosine methylation was sufficient to increase *HOXC10* expression in A549 cells. We treated A549 cells with a DNMT inhibitor, 5’Aza (1 μM) for 72 hrs. The mRNA levels of *HOXC10* were stimulated to a 3.5-fold increase by 5-Aza-CdR over the vehicle DMSO treated group in A549 cells (Figure 5B). These findings indicate a critical role for cytosine methylation in upregulation of *HOXC10* expression in NSCLC cells.

### G-quadruplex-mediated regulation of *HOXC10*

**Figure 6:**
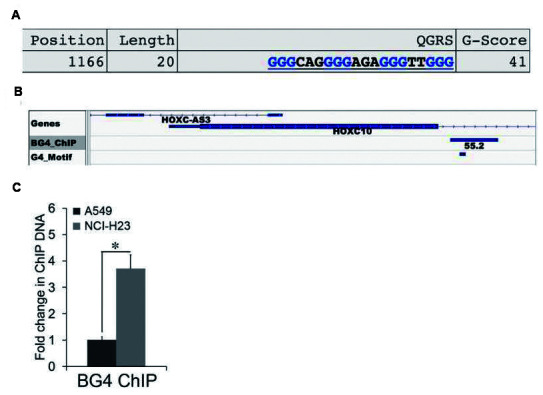
**Association between G-quadruplex and the expression of *HOXC10*.** (**A**) One G4 motif was identified by QGRS Mapper in the *HOXC10* CpG island. The sequences, length, and position of the G4 motif were illustrated. (**B**) The BED file of a genome-wide profiling of G4 formation using BG4-ChIP SEQ was extracted and analyzed. The G4 track was visualized using IGV Genome Browser. The G4 peak called within the HOXC10 CpG island was illustrated. The G4 motif identified by QGRS was marked within the G4 peak. (**C**) Formation of G4 within the *HOXC10* CpG island was assessed by ChIP assays using a G4-specific antibody BG4 in A549 and NCI-H23 cells. A fold change of the BG4 associated G4 region in NCI-H23 cells over A549 cells was obtained by setting the values from 2D culture to one. When presented, means and standard deviations were obtained from at least 3 independent experiments. * indicates a P value < 0.05.

CpG islands are frequently enriched with G4s [11]. Thus, we searched for G4 motifs in the *HOXC10* CpG island using QGRS Mapper, an algorithm that identifies G4 forming sequences [24]. We identified a G4 motif in the *HOXC10* CpG island using the formula [G3N1–7G3N1–7 G3N1–7 G3] (Figure 6A). Formation of G-quadruplex by the G4 motif in the *HOXC10* CpG island was supported by survey of a recent genome wide sequencing of G4 formation by ChIP assays with a G4-specific antibody BG4 coupled with next generation sequencing (BG4 ChIP-SEQ) (SRA# SRP068243) [25]. A BG4 ChIP peak that was called in the *HOXC10* CpG island harbored the G4 motif identified by QGRS Mapper (Figure 6B). We then employed BG4 ChIP to compare G4 formation in the *HOXC10* CpG island between A549 and NCI-H23 cells (Figure 6C). Indeed, the BG4 ChIP signal in the G4 harboring region was higher in the *HOXC10* high NCI-H23 cells than the *HOXC10* low A549 cells (Figure 6C).

**Figure 7:**
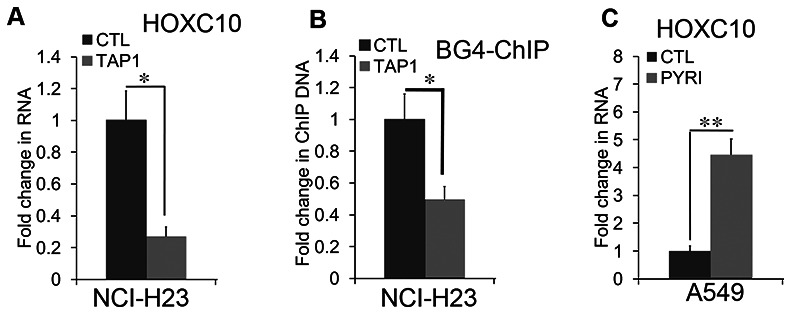
**G-quadruplex-mediated regulation of HOXC10 expression.** (**A**) NCI-H23 cells were exposed to the G4 disruptive ligand TAP1 (2 μM) for 4 days. Total RNA was extracted and assessed for the RNA levels of *HOXC10* using qRT-PCR. A fold change was obtained by normalizing to the housekeeping gene RPLP0 and setting the values from the DMSO control group to one. (**B**) Similar to part D except that the BG4 ChIP signals were compared between the DMSO control and TAP1 treated groups. (**C**) A549 cells were exposed to the G4 enhancing ligand pyridostatin (PYRI) (5 μM) for 48 hrs. Total RNA was extracted and assessed for the RNA levels of *HOXC10* using qRT-PCR. A fold change was obtained by normalizing to the housekeeping gene RPLP0 and setting the values from the DMSO group to one. When presented, means and standard deviations were obtained from at least 3 independent experiments. * and ** indicate a P value < 0.05 and 0.01, respectively.

To assess the role of G4 in the expression of *HOXC10*, we examined the effects of TAP1, a G4 disrupting ligand on the RNA levels of *HOXC10* in the *HOXC10* high NCI-H23 cells (Figure 7A). Exposure to TAP1 (2 μM) for 4 days resulted in a greater than 70% decrease in the RNA levels of *HOXC10* in NCI-H23 cells (Figure 7A). Consistently, TAP1 substantially reduced the BG4 ChIP signal in NCI-H23 cells (Figure 7B). In contrast, exposure to pyridostatin (5 μM), a G4 enhancing ligand for 48 hrs increased the RNA levels of *HOXC10* in *HOXC10* low A549 cells (7C). These findings suggest that G4 formation in the *HOXC10* CpG island mediates the expression of *HOXC10* in NSCLC cells.

## DISCUSSION

*HOXC10* is a critical gene that regulates the development of spinal cord, formation of neurons, and tumorigenesis [26–28]. Aberrant overexpression of *HOXC10* and its role in cancer progression has been reported in several types of cancers. However, the expression and functions of *HOXC10* in lung cancer remains unknown.

In the present study, we demonstrated that the expression *HOXC10* was upregulated in the aggressive NCI-H23 cells and modestly expressed in the well-differentiated A549 cells. Elevated expression of *HOXC10* was associated with poor overall and disease-free survival in the TCGA Lung Adenocarcinoma cohort (Figure 1D, 1E). These findings indicated clinical significance of elevated expression of *HOXC10* in lung cancer. Our analyses of *HOXC10* function in lung cancer further supported this notion. The clinical significance of *HOXC10* in NSCLC was further supported by two recent studies that relate elevated *HOXC10* expression to poor prognosis and disruption of epigenetic staging in NSCLC [29, 30]. Silencing *HOXC10* expression significantly repressed proliferation and promoted apoptosis in NCI-H23 cells. In contrast, forced overexpression of *HOXC10* promoted cell growth and decreased apoptosis in the *HOXC10* low A549 cells. More importantly, *HOXC10* overexpression in A549 cells was sufficient to promote tumor growth in vivo. We also observed altered expression of the apoptosis-related genes in response to the overexpression of *HOXC10* in A549 cell line and knockdown of *HOXC10* in NCI-H23 cells. Bcl-2 is a proto-oncogene that inhibits apoptosis and promotes survival of cancer cells under deleterious condition [31]. Bax as a pro-apoptotic protein can initiate cell death pathways, although it has similar sequence homology with Bcl-2 [32]. The ratio of Bcl-2 and Bax determines the response to a death signal via modulating membrane permeability of transition pore opening. Our results indicated that overexpressed HOXC10 can inhibit A549 cell apoptosis by increasing the ratio of Bcl-2/Bax, whereas knockdown of HOXC10 in NCI-H23 cells decreased them.

Since we discovered that *HOXC10* plays a critical role in NSCLC cell proliferation and apoptosis, we aimed to dissect the molecular mechanism that upregulate the expression of *HOXC10* in NSCLC cells. We demonstrated that hypomethylation was one mechanism for aberrant overexpression of *HOXC10* in NSCLC cells. This is consistent with Kim’s study that suggested hypomethylation of *HOXC10* CpG sites was associated with overexpression of *HOXC10* in gastric cancer [6]. Moreover, Pathiraja et al. reported that hypermethylation of *the HOXC10* CpG island was associated with transcriptional silencing in breast cancer cells [7].

Several recent reports suggest a link between G4 and chromatin staging for active transcription, especially histone acetylation and cytosine hypomethylation [33, 34]. We identified a G4 motif in the *HOXC10* CpG island and postulated that G4 formation mediated upregulation of *HOXC10* expression in NSCLC cells (Figures 6, 7). Promoting G4 has been extensively investigated as a therapeutic approach in cancer because targeting G4s with small molecules has been reported to be a strategy to suppress the expression of oncogenes, such as *Myc*, *Kars* and *Kit* [35]. Development of G4 stabilizing/enhancing ligands for cancer therapy has been largely based on the premise that accumulation of G4 in the oncogenes’ promoters can silence the expression of oncogenes. However, our characterization of G4s in the *HOXC10* CpG island challenges this oversimplified paradigm. Our results indicated that G4 formation was critical for upregulation of *HOXC10* in NSCLC cells. This notion is based on our findings that enhancing G4 by the stabilizing ligands can increase the expression of *HOXC10*, whereas disrupting G4 by TAP1 can decrease the expression of *HOXC10* (Figures 6, 7). We have not identified any genetic difference in the *HOXC10* promoter and gene body between A549 and NCI-H23 cells based on our analyses of the available data. However, we speculate that G4 formation may activate gene expression via facilitating transcription factor binding and staging chromatin structure favorable for transcription as proposed in a recent review of the function of G4 in human biology [36]. The different G4 formation in the *HOXC10* gene between A549 and NCI-H23 may result from different activity of the enzymes responsible for G4 formation and disruption. Further analyses are needed to fully understand how G4 formation upregulates *HOXC10* expression in NSCLC cells. Therefore, it is imperative to thoroughly understand how G4 upregulates and down-regulates genes in a genomic context dependent manner. Given the link between G4 formation and transcriptionally active chromatin stage, our results warrant further investigation of coordination of these epigenetic codes in regulation of gene expression in lung cancer cells.

Overall, our study indicated a key role of *HOXC10* in the proliferation and apoptosis of NSCLC cells in vivo and in vitro. Our results suggested that *HOXC10* promoted survival via upregulation of Bcl-2. Moreover, we revealed a new mechanism mediated by DNA demethylation and G4 formation in promoting *HOXC10* expression and lung cancer development. Thus, *HOXC10* emerges as novel therapeutic target due to its regulation of proliferation and survival and a target of G4 disrupting agents that are well advanced in preclinical and clinical anticancer research.

## MATERIALS AND METHODS

### Reagents and plasmids

5-Aza-2′-deoxycytidine (5-Aza-2dC), a small molecular inhibitor of DNA methyltransfereases (DNMT), was purchased from BioVision (Milpitas, CA). A HOXC10-specific antibody (ab15390) was purchased from Abcam (Cambridge, MA). A Bcl-2-speicfica antibody (12789-1-AP) and a Bax-specific antibody (60267-1-Ig) were purchased from Proteintech Group (Chicago, IL). Pyridostatin, a G4 stabilizing ligand was obtained from Cayman (Ann Arbor, MI). TAP1, a G4 disrupting ligand, was kindly provided by Dr. Shankar Balasubramanian at University of Cambridge [37]. Sequences of the primers and siRNAs were listed in Supplementary Table 1.

### Cell culture

Human non small lung cancer cell lines A549 and NCI-H23 were purchased from ATCC and cultured in RPMI1640 as we described elsewhere (Manassas, VA) [38].

### Transfection

The human HOXC10-specific siRNAs were purchased from GenePharma (Shanghai, China). All the siRNAs were transfected at 60 nM into NCI-H23 cells using RNAiMAX according to the reverse transfection protocol provided by the vendor (Invitrogen, Carlsbad CA) [39].

### Generation of HOXC10 stable A549 variants

The constructed HOXC10 overexpression plasmids were transfected into 293 T cells, together with package plasmids pCDH-GFP (pCDH-HOXC10), delta8.9 and VSVG at the ration of 15:12:8 using Transfection Reagent Lipofectamine 2000 (Invitrogen). The lentivirus particles containing supernatants were collected and filtered through 0.4 μm filter membrane after 72 h. A549 cells were plated in 6-well plates with 1 × 10^6^ cells per well and transfected by collected lentivirus particles. The transfected cells were selected by puromycin for 2 weeks. Establishment of stable HOXC10 overexpressing A549 cells was verified by Western blot.

### RNA extraction and quantitative RT-PCR

Total cell RNA was extracted using Trizol per the protocol provided by the vendor (Invitrogen, Carlsbad CA) as we previously described (33). The RNA levels of each gene were quantified using qRT-PCR on StepOne Plus Thermal Cycler (Invitrogen, Carlsbad CA) and compared among the groups using the delta CT method [40]. A fold change of each RNA transcript was obtained by normalizing to the house keep gene GAPDH and setting the values from the control group to one.

### Cell proliferation and viability assay

Cell viability was assessed when *HOXC10* was overexpressed in A549 cells and knockdown in NCI-H23 cells, respectively. Cell viability was assessed using MTT Assays per the provider’s instructions (ThermoFisher, Waltham, MA) [41].

### Assessment of apoptosis

Cell apoptosis was evaluated by flow cytometry using AnnexinV FITC Apoptosis Detection Kit according to the manufacturer’s instructions (Vazyme Biotech Co., Nanjing, China). Briefly, the cells (1×10^5^) were harvested and washed twice in phosphate-buffered saline (PBS) and resuspended in 500 μL of binding buffer and then stained in 10 μL of Annexin V and 5-μL propidium iodide (PI) and analyzed using a flow cytometer (Becton Dickinson, Franklin Lakes, NJ, USA).

### RNA-SEQ analysis

In house RNA-SEQ data were generated from human NSCLC cell lines A549 and H23 at Washington State University Spokane Genomic Core. Libraries were constructed using the Illumina TruSeq Stranded Total RNA Library Prep Kit/Ribo-Zero Gold kit. RNA-SEQ was carried out on ILLUMINA HISEQ 2500. Roughly 40 million paired end stranded 100 bp reads were generated from each RNA sample. The RNA-SEQ data can be accessed at NCBI GEO (GSE119513). All RNA-SEQ data were analyzed using the Star-RSEM-EbSeq pipeline. Three biological replicates from each cell line were included in our analyses. A transcript was defined as differentially expressed between A549 and NCI-H23 cells when its difference between two cell lines was greater than 2-fold and its false discovery rate (FDR) was smaller than 0.01.

### BS-SEQ analysis

Raw whole-genome bisulfite sequencing (BS-SEQ) reads from MCF-7, MDA-MB-468, T47D, and MDA-MB-231 cells were obtained from the NCBI Sequence Read Archive (SRA# SRP005601). The BS-SEQ data was analyzed using methylKit [42]. Only the cytosine sites with a combined reads of methylated and unmethylated cytosine greater than 10 and below the 99.9^th^ percentile were included in our analyses.

### Implantation of lung cancer cells

All mouse studies were carried out following the animal protocol approved by the Institute Animal Care and Use Committee at Wuhan Servivebio. Subcutaneous implantation of human NSCLC cell line A549 cells (2 × 10^6^cells/mouse) into male nude and syngeneic mice was carried out as we previously described [43]. Each group of tumor graft consisted of 7 mice. Tumor growth was monitored daily after implantation. The tumor mass was dissected from mice at four weeks after implantation and processed for weighing and H&E staining.

### Chromatin immunoprecipitation for G-quadruplex motifs

Chromatin Immunoprecipitation (ChIP) assay was employed to assess G4 formation in the *HOXC10* CpG island region. BG4, a G4-specific antibody was purchased from Millipore and used in ChIP assays to immunoprecipitated G4 motifs as described elsewhere [25].

### Immunoblot

Total cell lysates were extracted from A549 and H23 cells as well as their variants with HOXC10 overexpressed or knockdown. Immunoblotting was used to assess the protein levels of the indicated genes and the loading control gene GAPDH [44].

### Statistical analysis

When presented, means and standard deviations were obtained from at least three independent experiments. A P value between any two compared groups was determined using unpaired two-tailed Student’s t-test.

## Supplementary Material

Supplementary Figure 1

Supplementary Table 1
