# Supplementary material for: Epigenetic upregulation of HOXC10 in non-small lung cancer cells
Source: Aging (Albany NY). 2020 Jul 19;12(17):16921–35. doi: 10.18632/aging.103597 (PMC7521540; doi:10.18632/aging.103597)
Supplement: Supplementary Figure 1 [file aging-12-103597-s002..pdf]

SUPPLEMENTARY FIGURE

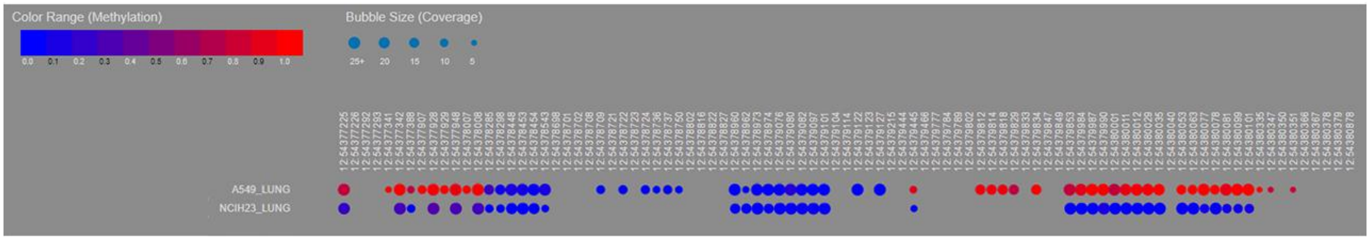

Supplementary Figure 1. Methylation profiles in the HOXC10 CpG island between A549 and NCI-H23 cells.
