# Supplementary material for: Epigenetic upregulation of HOXC10 in non-small lung cancer cells
Source: Aging (Albany NY). 2020 Jul 19;12(17):16921–35. doi: 10.18632/aging.103597 (PMC7521540; doi:10.18632/aging.103597)
Supplement: Supplementary Table 1 [file aging-12-103597-s001..pdf]

SUPPLEMENTARY TABLE

Supplementary Table 1. Sequences of the primers and siRNAs.

Gene name		5'-3'Sequence	size
HOXC10	Forward	ACGAAGCGAAAGAGGAGATAAAG	105bp
	Reverse	CCAGCGTCTGGTGTTTAGTATAG	
GAPDH	Forward	TCAAGAAGGTGGTGAAGCAGG	115bp
	Reverse	TCAAAGGTGGAGGAGTGGGT	
HOXC10-homo-397	Forward	CCUACCCACCUAGUGUCAATT	
(siRNA1)	Reverse	UUGACACUAGGUGGGUAGGTT	
HOXC10-homo-876	Forward	GGAAAUUGGCUGACAGCAATT	
(siRNA2)	Reverse	UUGCUGUCAGCCAAUUUCCTT	
HOXC10-homo-995	Forward	GGAGAUUAGCAAGACCAUUTT	
(siRNA3)	Reverse	AAUGGUCUUGCUAAUCUCCTT	
